# COVLIAS 1.0 vs. MedSeg: Artificial Intelligence-Based Comparative Study for Automated COVID-19 Computed Tomography Lung Segmentation in Italian and Croatian Cohorts

**DOI:** 10.3390/diagnostics11122367

**Published:** 2021-12-15

**Authors:** Jasjit S. Suri, Sushant Agarwal, Alessandro Carriero, Alessio Paschè, Pietro S. C. Danna, Marta Columbu, Luca Saba, Klaudija Viskovic, Armin Mehmedović, Samriddhi Agarwal, Lakshya Gupta, Gavino Faa, Inder M. Singh, Monika Turk, Paramjit S. Chadha, Amer M. Johri, Narendra N. Khanna, Sophie Mavrogeni, John R. Laird, Gyan Pareek, Martin Miner, David W. Sobel, Antonella Balestrieri, Petros P. Sfikakis, George Tsoulfas, Athanasios Protogerou, Durga Prasanna Misra, Vikas Agarwal, George D. Kitas, Jagjit S. Teji, Mustafa Al-Maini, Surinder K. Dhanjil, Andrew Nicolaides, Aditya Sharma, Vijay Rathore, Mostafa Fatemi, Azra Alizad, Pudukode R. Krishnan, Ferenc Nagy, Zoltan Ruzsa, Archna Gupta, Subbaram Naidu, Kosmas I. Paraskevas, Mannudeep K. Kalra

**Affiliations:** 1 Stroke Diagnostic and Monitoring Division, AtheroPoint™, Roseville, CA 95661, USA; drindersingh1@gmail.com (I.M.S.); pomchadha@gmail.com (P.S.C.); 2 Advanced Knowledge Engineering Centre, Global Biomedical Technologies, Inc., Roseville, CA 95661, USA; sushant.ag09@gmail.com (S.A.); samriddhiagarwal12@gmail.com (S.A.); lakshayjgupta@gmail.com (L.G.); 3 Department of Computer Science Engineering, Pranveer Singh Institute of Technology, Kanpur 209305, India; 4 Department of Radiology, “Maggiore della Carità” Hospital, University of Piemonte Orientale (UPO), 28100 Novara, Italy; profcarriero@virgilio.it; 5 Department of Radiology, Azienda Ospedaliero Universitaria (A.O.U.), 09124 Cagliari, Italy; pascheale@gmail.com (A.P.); psc.dnn@gmail.com (P.S.C.D.); martagiuliacol@gmail.com (M.C.); lucasabamd@gmail.com (L.S.); antonellabalestrieri@hotmail.com (A.B.); 6 Department of Radiology and Ultrasound, University Hospital for Infectious Diseases, 10 000 Zagreb, Croatia; klaudija.viskovic@bfm.hr (K.V.); mehmedovic.armin302@gmail.com (A.M.); 7 Department of Pathology, AOU of Cagliari, 09124 Cagliari, Italy; gavinofaa@gmail.com; 8 The Hanse-Wissenschaftskolleg Institute for Advanced Study, 27753 Delmenhorst, Germany; monika.turk84@gmail.com; 9 Department of Medicine, Division of Cardiology, Queen’s University, Kingston, ON K7L 3N6, Canada; johria@queensu.ca; 10 Department of Cardiology, Indraprastha APOLLO Hospitals, New Delhi 110076, India; drnnkhanna@gmail.com; 11 Cardiology Clinic, Onassis Cardiac Surgery Center, 17674 Athens, Greece; soma13@otenet.gr; 12 Heart and Vascular Institute, Adventist Health St. Helena, St Helena, CA 94574, USA; Lairdjr@ah.org; 13 Minimally Invasive Urology Institute, Brown University, Providence, RI 02912, USA; gyan_pareek@brown.edu (G.P.); dwsobel@gmail.com (D.W.S.); 14 Men’s Health Center, Miriam Hospital, Providence, RI 02906, USA; martin_miner@brown.edu; 15 Rheumatology Unit, National Kapodistrian University of Athens, 15772 Athens, Greece; psfikakis@med.uoa.gr; 16 Department of Surgery, Aristoteleion University of Thessaloniki, 54124 Thessaloniki, Greece; tsoulfasg@gmail.com; 17 Cardiovascular Prevention and Research Unit, Department of Pathophysiology, National & Kapodistrian University of Athens, 15772 Athens, Greece; aprotog@med.uoa.gr; 18 Department of Immunology, Sanjay Gandhi Postgraduate Institute of Medical Sciences, Lucknow 226014, India; durgapmisra@gmail.com (D.P.M.); vikasagr@yahoo.com (V.A.); 19 Academic Affairs, Dudley Group NHS Foundation Trust, Dudley DY1 2HQ, UK; george.kitas@nhs.net; 20 Arthritis Research UK Epidemiology Unit, Manchester University, Manchester M13 9PL, UK; 21 Ann and Robert H. Lurie Children’s Hospital of Chicago, Chicago, IL 60611, USA; jteji@mercy-chicago.org; 22 Allergy, Clinical Immunology and Rheumatology Institute, Toronto, ON L4Z 4C4, Canada; almaini@hotmail.com; 23 AtheroPoint LLC, Roseville, CA 95611, USA; surinderdhanjil@gmail.com (S.K.D.); Vijay.s.rathore@kp.org (V.R.); 24 Vascular Screening and Diagnostic Centre and University of Nicosia Medical School, Nicosia 2408, Cyprus; anicolaides1@gmail.com; 25 Division of Cardiovascular Medicine, University of Virginia, Charlottesville, VA 22904, USA; AS8AH@hscmail.mcc.virginia.edu; 26 Department of Physiology & Biomedical Engg., Mayo Clinic College of Medicine and Science, Rochester, MN 55905, USA; fatemi.mostafa@mayo.edu; 27 Department of Radiology, Mayo Clinic College of Medicine and Science, Rochester, MN 55905, USA; Alizad.Azra@mayo.edu; 28 Neurology Department, Fortis Hospital, Bangalore 560076, India; prkrish12@rediffmail.com; 29 Internal Medicine Department, University of Szeged, 6725 Szeged, Hungary; drnagytfer@hotmail.com; 30 Invasive Cardiology Division, University of Szeged, 6725 Szeged, Hungary; zruzsa@icloud.com; 31 Radiology Department, Sanjay Gandhi Postgraduate Institute of Medical Sciences, Lucknow 226014, India; garchna@gmail.com; 32 Electrical Engineering Department, University of Minnesota, Duluth, MN 55812, USA; dsnaidu@d.umn.edu; 33 Department of Vascular Surgery, Central Clinic of Athens, 14122 Athens, Greece; paraskevask@hotmail.com; 34 Department of Radiology, Massachusetts General Hospital, Boston, MA 02114, USA; MKALRA@mgh.harvard.edu

**Keywords:** COVID-19, CT, lung segmentation, COVLIAS, MedSeg, AI, DL, HDL, validation, benchmark.4

## Abstract

(1) Background: COVID-19 computed tomography (CT) lung segmentation is critical for COVID lung severity diagnosis. Earlier proposed approaches during 2020–2021 were semiautomated or automated but not accurate, user-friendly, and industry-standard benchmarked. The proposed study compared the COVID Lung Image Analysis System, COVLIAS 1.0 (GBTI, Inc., and AtheroPoint^TM^, Roseville, CA, USA, referred to as COVLIAS), against MedSeg, a web-based Artificial Intelligence (AI) segmentation tool, where COVLIAS uses hybrid deep learning (HDL) models for CT lung segmentation. (2) Materials and Methods: The proposed study used 5000 ITALIAN COVID-19 positive CT lung images collected from 72 patients (experimental data) that confirmed the reverse transcription-polymerase chain reaction (RT-PCR) test. Two hybrid AI models from the COVLIAS system, namely, VGG-SegNet (HDL 1) and ResNet-SegNet (HDL 2), were used to segment the CT lungs. As part of the results, we compared both COVLIAS and MedSeg against two manual delineations (MD 1 and MD 2) using (i) Bland–Altman plots, (ii) Correlation coefficient (CC) plots, (iii) Receiver operating characteristic curve, and (iv) Figure of Merit and (v) visual overlays. A cohort of 500 CROATIA COVID-19 positive CT lung images (validation data) was used. A previously trained COVLIAS model was directly applied to the validation data (as part of Unseen-AI) to segment the CT lungs and compare them against MedSeg. (3) Result: For the experimental data, the four CCs between COVLIAS (HDL 1) vs. MD 1, COVLIAS (HDL 1) vs. MD 2, COVLIAS (HDL 2) vs. MD 1, and COVLIAS (HDL 2) vs. MD 2 were 0.96, 0.96, 0.96, and 0.96, respectively. The mean value of the COVLIAS system for the above four readings was 0.96. CC between MedSeg vs. MD 1 and MedSeg vs. MD 2 was 0.98 and 0.98, respectively. Both had a mean value of 0.98. On the validation data, the CC between COVLIAS (HDL 1) vs. MedSeg and COVLIAS (HDL 2) vs. MedSeg was 0.98 and 0.99, respectively. For the experimental data, the difference between the mean values for COVLIAS and MedSeg showed a difference of <2.5%, meeting the standard of equivalence. The average running times for COVLIAS and MedSeg on a single lung CT slice were ~4 s and ~10 s, respectively. (4) Conclusions: The performances of COVLIAS and MedSeg were similar. However, COVLIAS showed improved computing time over MedSeg.

## 1. Introduction

COVID-19 lung segmentation in computed tomography (CT) scans is critical for determining lung severity [1,2,3]. According to the World Health Organization (WHO), as of 4 November 2021, more than 247 million individuals have been infected with the acute respiratory syndrome coronavirus 2 (SAR-COV-2). A fraction of the world’s population has come into contact with Acute Respiratory Distress Syndrome (ARDS), which has resulted in the death of 5 million people [4]. COVID-19 worsens with comorbidity, affecting other organs such as coronary artery disease, [5,6], diabetes [7], atherosclerosis [8], fetal [9], pulmonary embolism [10], and stroke [11]. In patients with underlying comorbidities or moderate to severe disease, chest radiographs and CT [12,13,14] are utilized to identify acute ARDS severity based on the number of pulmonary opacities such as ground-glass (GGO), consolidation, and mixed [2,15,16,17]. To describe the severity of COVID-19 pneumonia, most radiologists provide a semantic description of the degree and its kind of opacities. These methods are time-consuming and subjective for the examination of pulmonary opacities [18,19,20,21]. As part of the pipeline for COVID-19 diagnosis, CT lung segmentation is crucial [1,2,3]. Here is where artificial intelligence (AI) comes into play in automating this time-consuming process and providing a faster diagnosis of the disease [22,23,24,25].

**Figure 1:**
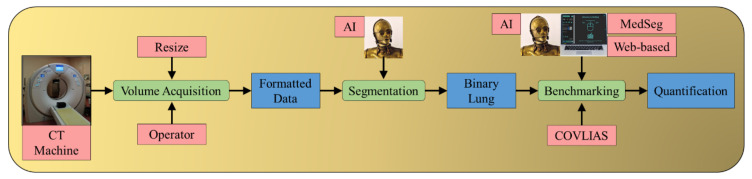
Pipeline for comparing AI-based COVLIAS and MedSeg. The benchmarking stage shows the comparison between COVLIAS and MedSeg. The CT machine uses the ITALIAN cohort during experimentation, and during validation, the CT machine uses the CROATIA cohort.

COVLIAS 1.0 (Figure 1) [1] is a global lung segmentation and evaluation system using AI-based segmentation models. This robust AI system (from here on will be called COVLIAS) is designed to keep in mind its clinical acceptability for the segmentation of COVID-19 affected CT lungs. The proposed study presents a comparison of COVLIAS [1,2,3] and the previously offered AI integrated web-based CT image segmentation tool MedSeg [26]. The working of COVLIAS and MedSeg is discussed briefly in the methodology section. The performance evaluation section shows the comparison of COVLIAS and MedSeg on ITALIAN experimental data using (i) Bland–Altman (BA) plots, (ii) correlation coefficient (CC) plots, (iii) Receiver operating characteristic (ROC) curve, and (iv) Figure of Merit (FoM). Further, we used validation data from CROATIA consisting of COVID-19 positive CT lung images to compare COVLIAS vs. MedSeg. These validation data were never seen by the AI model. They are categorized as unseen-AI, where the dataset was taken from a different clinical setting, making it perfect for validating the COVLIAS against MedSeg systems.

The layout of this comparative study is as follows: Section 2 discusses the background literature. Section 3 presents the methodology, where we discuss the demographics and AI architectures. The results and performance evaluation are discussed in Section 4. The comparison between our study and similar studies is presented in the benchmarking table in Section 5. The same section also discusses the strengths, weaknesses, and extensions. The study concludes in Section 6.

## 2. Background Literature

The concept of utilizing AI to characterize diseases has been implemented in almost all areas of medical imaging. This includes the AI’s function in locating the disease, extracting the disease’s region of interest, and automatically classifying the disease against binary or multiclass events. We chose characterization methods based on machine learning and deep learning that overlap and synchronize with ARDS frameworks. The purpose of adopting this characterization system is to communicate the origination and innovation spirits that have been derived for various modalities, organs, and applications. Examples of AI-based characterization can be seen in several other disease diagnosis applications such as brain [27,28,29], stroke [30,31,32], liver [33,34,35], coronary artery [36,37], prostate [38], ovarian [39,40], diabetes [41], thyroid cancer [42], skin cancer [43,44], and heart [45,46,47]. This framework can also be extended to the COVID-19 framework.

COVID-19 is genetically similar to SARS-CoV-1, but not the coronavirus that causes Middle East respiratory syndrome (MERS-CoV). The incubation duration, clinical severity, and transmissibility are all different from SARS-CoV-1 [48]. The global spread of COVID-19 has expanded despite government measures to establish social habits such as social distancing and wearing masks and quarantining and non-pharmacological, preventive treatments for psychophysical well-being [49,50]. To describe the severity of COVID-19 pneumonia, most radiologists provide a semantic description of the degree and kind of opacities. The semiquantitative assessment of pulmonary opacities is time-consuming, subjective, and labor-intensive [18,19,20,21]. In disease detection, segmentation [51,52,53,54] and classification [47,55] are two primary components, with segmentation playing a critical role. Deep learning (DL), an machine learning (ML) extension, uses thick layers to automatically extract and classify all important imaging characteristics [23,56,57,58,59,60]. Hybrid DL (HDL), an approach that integrates two AI systems, aids in addressing some of the issues encountered with DL models, such as overfitting and optimizing hyperparameters to remove bias [61].

## 3. Material and Research Methodology

### 3.1. Material: Patient Demographics and Image Acquisition

#### 3.1.1. Demographics for Italian and Croatian Databases

The dataset includes 72 adult Italian patients (experimental database), 46 of them are male, and the rest are female. The average height in males and females is 171 cm, and 175 cm, respectively, and the average weight in males and females is 76 kg and 83 kg, respectively. A total of 60 individuals tested positive for RT-PCR, with bronchoalveolar lavage confirming 12 of them [62]. The cohort had an average GGO of 4.1 and consolidation of 2.4, which was regarded as mild. Following are the percentage distribution out of the 72 individuals having cough (45%), sore throat (8%), dyspnea (54%), chronic inflammatory lung disease (COPD) (42%), hypertension (12.5%), diabetes (11%), smokers (11%), and cancer (14%).

The validation cohort consisted of 500 CT scans (validation database, taken from 7 patients from Croatia) with a mean age of 66 (SD 7.988), 5 of them male (71.4%) and the rest female. The average GGO and consolidation scores were 2 and 1.2 in the cohort, respectively. Out of 7 selected patients in this study, all of them had a cough, 85% were reported to have dyspnea and hypertension (28%), 14% were smokers, and none of them had a sore throat or diabetes, COPD, and cancer. None of them were admitted to the Intensive Care Unit (ICU), and none of the patients died due to COVID-19.

#### 3.1.2. Image Acquisition for Italian and Croatian Cohorts

**Figure 2:**
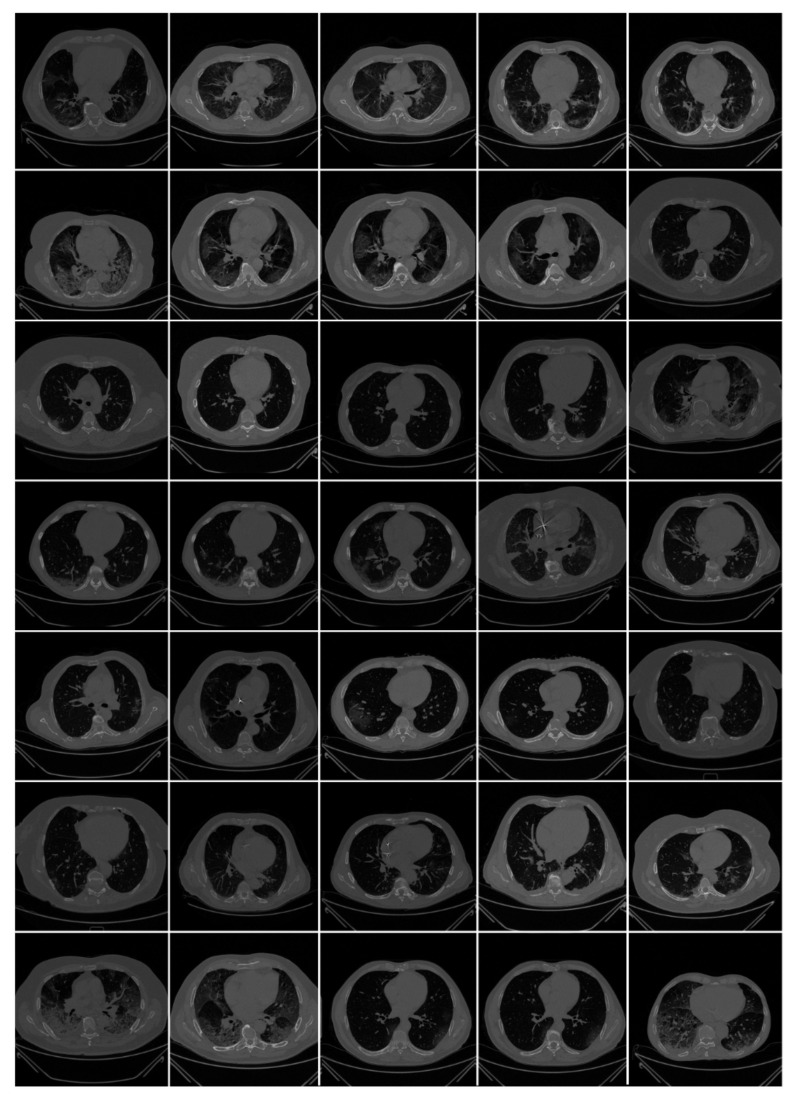
Raw CT images from NOVARA, ITALIAN dataset [3].

*Italian cohort*: All chest CT scans were performed in a supine position with a single full inspiratory breath-hold, utilizing a 128-slice multidetector-row “Philips Ingenuity Core” CT scanner from Philips Healthcare(Netherlands). There was no intravenous or oral injection of contrast media. A soft tissue kernel with 512 × 512 matrix (mediastinal window) and a lung kernel with 768 × 768 matrix (lung window) were utilized to rebuild one-mm thick pictures. The CT tests were carried out with a 120 kV, 226 mAs/slice (using Philips’ automatic tube current modulation—Z-DOM), 1.08 spiral pitch factor, 0.5-s gantry rotation time, and 64 × 0.625 detector configuration. The CT data of 72 COVID-positive individuals were used in the proposed investigation. CT volumes of patients were selected based on two criteria (i) the image quality should be reasonable and should have no artifacts or blurriness due to body movement, and (ii) there is no metallic object present in the scan area. Each patient consisted of approximately 200 slices from which the radiologist [LS] selected 65–70 slices from the visible lung region (Figure 2), yielding a total of 5000 images. These 5000 images were used to train and test AI-based segmentation models in the COVLIAS 1.0 system.

**Figure 3:**
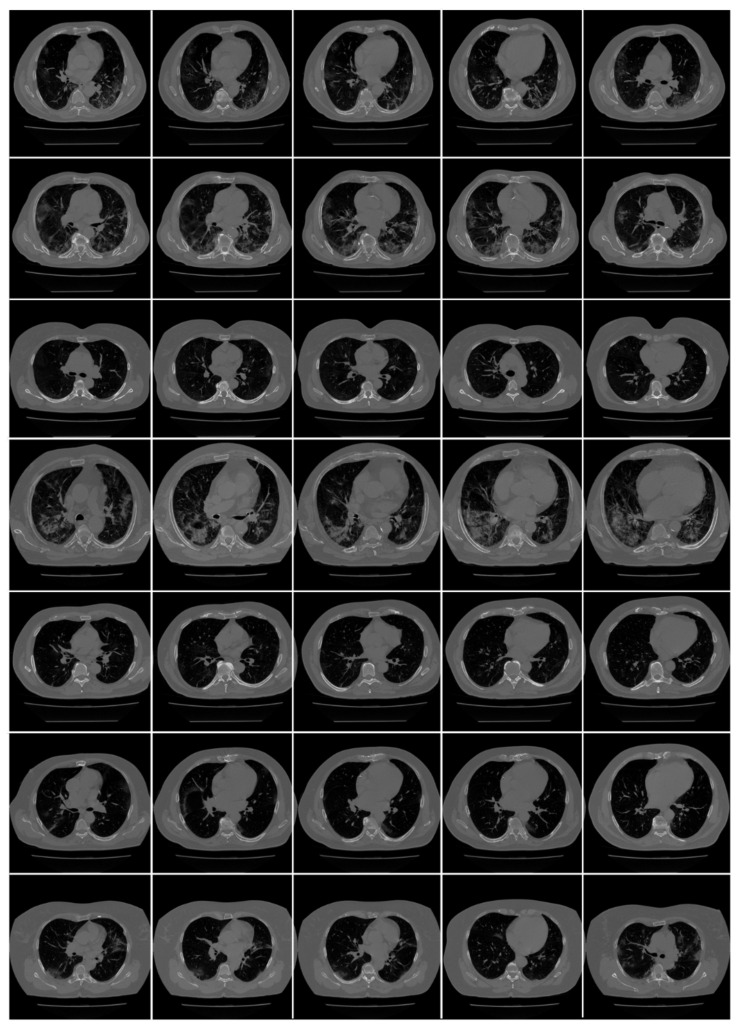
Raw CT images from the CROATIA dataset.

*Croatian Cohort*: A CROATIAN patient of seven COVID-19 positive patients (500 images) was used to validate the AI system (COVLIAS). All chest multidetector CT images (MDCT) were performed in a supine position with a single full inspiratory breath-hold utilizing FCT Speedia HD (Fujifilm Corporation, Tokyo, Japan, 2017) 64-detector MDCT scanner to acquire images of the thorax in craniocaudal direction. Images were acquired with a standard algorithm and viewed with Hitachi, Ltd. Whole Body X-ray CT System Supria Software (System Software Version: V2.25, Copyright Hitachi, Ltd. 2017). There was no contrast media available for intravenous or oral administration. The used scanned parameters were: volume scan, large focus, tube voltage 120 kV, tube current 350 mA with automatic tube current modulation mode (IntelliEC mode), and rotation speed 0.75 s. Parameters used for reconstruction were: field of view (FOV) 350 mm, slice thickness 5 mm (0.625 × 64), table pitch 1.3281, picture filter 32 with multi recon option: picture filter 22 (lung standard) with Intelli IP Lv.2 iterative algorithm (WW1600/WL600), slice thickness 1.25 mm, recon index 1 mm and picture filter 31 (mediastinal) with Lv.3 Intelli IP iterative algorithm (WW450/WL45), slice thickness 1.25 mm, recon index 1 mm. CT volumes of patients were selected based on two criteria (i) the image quality should be reasonable and should have no artifacts or blurriness due to body movement and (ii) there is no metallic object present in the scan area. Figure 3 shows an example of raw CT images from the cohort.

#### 3.1.3. Data Preparation

We follow the CT slice selection guidelines as used in our previous published studies [1,63,64,65], where the focus was to exclude the non-lung anatomy while preserving the most volumetric region of the lung. The idea was to get the entire visible lung region in each CT slice. Since the lung region (area) was only about ~20% of the whole CT image slice (768 × 768 px2), this accounted for the removal of nearly ~32% of the CT slices, each from the top and the bottom of the CT volume. It was equivalent to removing 65 CT slices from the top and bottom of the CT volume. Thus, the radiologist [LS] choose the remaining ~70 CT slices out of ~200 CT slices for each patient corresponding to the visible lung region.

### 3.2. Research Methodology and Experimental Protocol

#### 3.2.1. AI Architecture for Two Hybrid Models

**Figure 4:**
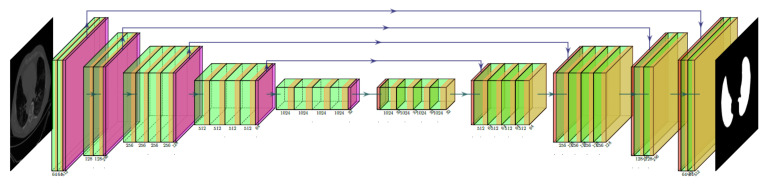
VGG-SegNet (HDL 1) architecture.

**Figure 5:**
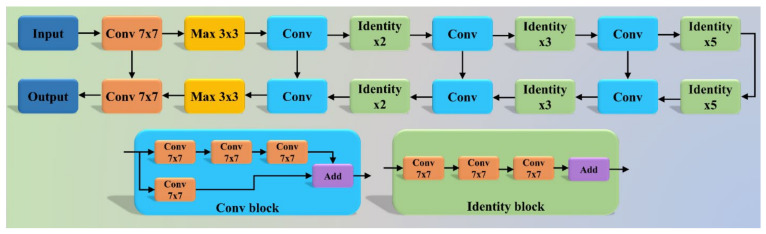
ResNet-SegNet (HDL 2) architecture.

The COVLIAS system incorporates two hybrid DL (HDL) (a) VGG-SegNet (HDL 1) and (b) ResNet-SegNet (HDL 2). The VGG-SegNet architecture (Figure 4) employed in this study is made up of three components: an encoder, a decoder, and a pixel-wise SoftMax classifier in the end. In comparison to the SegNet [66] architecture, it has 16 convolutions (Conv) layers (VGG backbone). In ResNet-SegNet (Figure 5), the difference is in the encoder and decoder parts. It is replaced with a ResNet [67] architecture. In this architecture, a new link known as the skip connection was developed, allowing gradients to bypass a specific number of levels to overcome the vanishing gradient problem.

#### 3.2.2. Loss Function Design

We adapted cross-entropy (CE)-loss during the training of the (a) VGG-SegNet (HDL 1) and (b) ResNet-SegNet (HDL 2) models. If α_i_ represents the input manual delineation label 1 (lung region), (1 − α_i_) represents the manual delineation label 0 (non-lung region), $p_{i}\;$ represents the probability of the HDL models (SoftMax) adapted at the prediction layer of the AI model, and the product is represented by × term and the symbol $L_{CE}$ represents CE-loss function, then $L_{CE}$ is mathematically represented, as shown in Equation (1).

(1) $$L_{CE}=-[(\alpha_{i}{\;\times \;\log\;p}_{i})+(1\;-\;\alpha_{i}{)\;\times \;\log(1\;-\;p}_{i})]$$

#### 3.2.3. Experimental Protocol

The AI models’ accuracy was determined using the standardized cross-validation (CV) technique. Because the data had mild COVID, the 5-fold (K5) CV procedure was utilized. In this experimental protocol, 4000 CT images (80% data) were used for training the two AI-based HDL models. The remaining 1000 CT images (20% remaining data) were used to test the performance of the AI models. During the K5 implementation, we ensured that every test fold was unique and mutually exclusive. Further, we ensure that 10% of training data is used to validate the AI system.

#### 3.2.4. Cross-Validation Accuracy

The output of the AI model in the foreground (white) region represents the segmented lung. The manual delineated region follows a similar setup where the foreground (white) region represents the MD lung. In the above scenarios, the foreground lung (white) region is represented a binary 1 and the background as binary 0. The accuracy of the HDL system is computed using the standardized formula, given the 2 × 2 truth table values, namely, true-positive (TP), true-negative (TN), false-negative (FN), and false-positive (FP). It can be mathematically represented in Equation (2).

(2) $$\mathrm{Accuracy}\;\left(\mathrm{HDL}\right)\;\left(\%\right)=\left(\frac{\mathrm{TP}+\mathrm{TN}}{\mathrm{TP}+\mathrm{FN}+\mathrm{TN}+\mathrm{FP}}\right)\times 100$$

#### 3.2.5. Lung Area Calculation and Figure of Merit

We calculate the area of the two balloon-shaped lungs using the foreground part of the binary image corresponding to the two AI systems. If *A*(*p*, *q*) represents the lung area for in the image “*q*” using model “*p*”, where “*p*” can take VGG-SegNet (HDL 1) and ResNet-SegNet (HDL 2). We adopt a resolution factor of 0.52 to convert pixel to mm^2^. Then the mean area for the HDL model “*p*” in mm^2^ is symbolized as ${\bar{A}}_{HDL}\left(p\right)\;$and can be mathematically represented using Equation (3).

(3) $${\bar{A}}_{HDL}\left(p\right)=\frac{\sum_{q=1}^{N}A\left(p,q\right)}{N}$$

We have used FoM to represent the AI systems error, if ${\bar{A}}_{MDr}$ represents a mean area of the manual delineated lungs, “*r*” represents the MD 1 or MD 2, then FoM for model “*p*” can be mathematically represented using Equation (4).

(4) $$FoM\left(p\right)=100-\left[\left(\frac{\left|{\bar{A}}_{HDL}\left(p\right)-{\bar{A}}_{MDr}\right|}{{\bar{A}}_{MD}}\right)\times 100\right]$$

#### 3.2.6. Performance Evaluation Criteria

As part of the performance evaluation criteria, we compare COVLIAS (HDL 1) vs. MD 1, COVLIAS (HDL 2) vs. MD 1, COVLIAS (HDL 1) vs. MD 2, COVLIAS (HDL 2) vs. MD 2, and COVLIAS vs. MedSeg using the following attributes (i) creating the BA plots, (ii) estimating the CC plots, (iii) ROC curve, and (iv) computing the FoM. Further, we use validation data from CROATIA consisting of COVID-19 positive CT lung images to compare COVLIAS vs. MedSeg.

#### 3.2.7. MedSeg—A Web-Based AI Segmentation Tool

**Figure 6:**
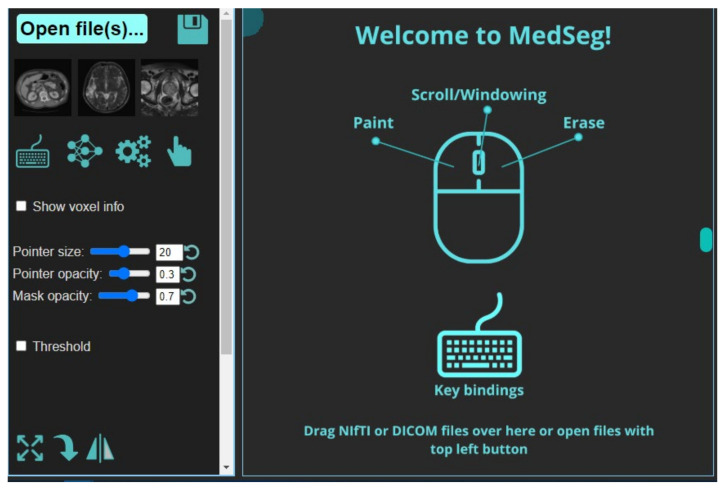
Opening page of the MedSeg tool.

**Figure 7:**
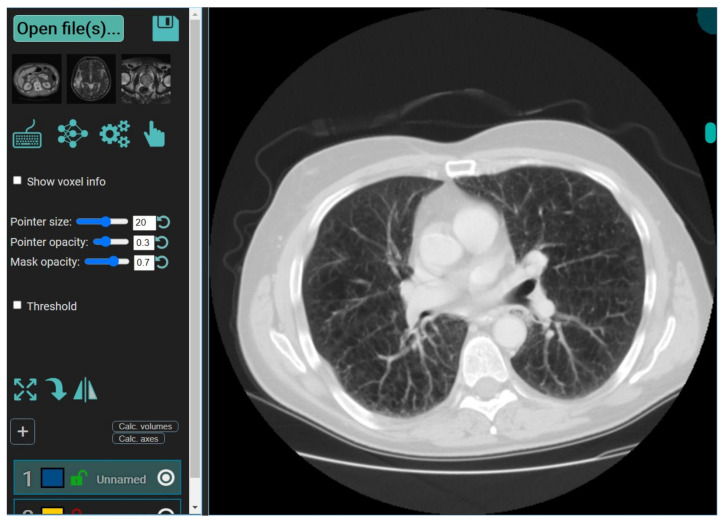
Display of CT image using the MedSeg tool.

**Figure 8:**
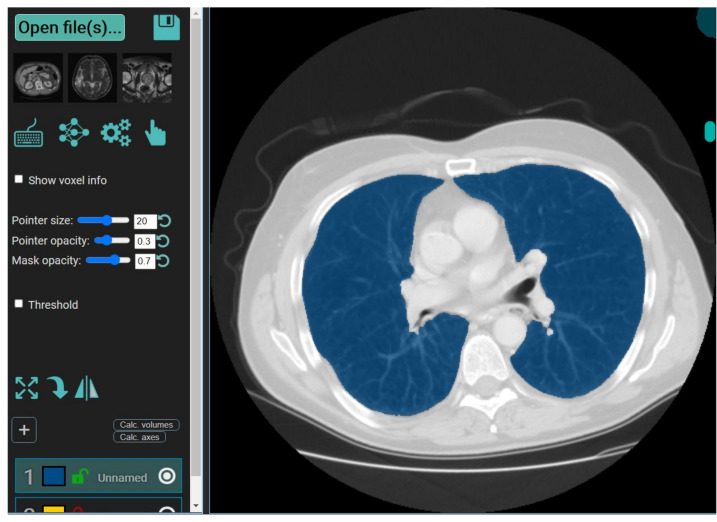
Segmentation of the lung in CT slice using the MedSeg tool.

It is a web-based annotation and segmentation tool for medical organs. The steps for segmentation include (i) Drag and dropping the collection of DICOM- files or single NIfTI-file into the segmentation zone as shown below. This is done after launching the MedSeg link. An alternate way is to click the upload button on the top left (Figure 6). If multiple DICOM-slices are uploaded, this tool will automatically stack them in the correct order. (ii) If one uses the computer’s central processing unit (CPU) that has relatively up-to-date hardware, then it will give the best experience in terms of avoiding lag. (iii) Once the image is loaded, it will appear with a default window level/gray scaling (Figure 7). (iv) Next, select the CT lung segmentation model from the list for segmentation of the lung. (v) It will show the line “CT Thorax lungs model loaded” and activate the segmentation process (Figure 8). (vi) Scroll images using the mouse wheel or press ‘Up arrow’ and ‘Down arrow’ to move one slice at a time. (vii) After the segmentation process is complete, click the save icon. The final result will be a NIfTI [68] file with the annotated mask.

## 4. Results and Performance Evaluation

**Figure 9:**
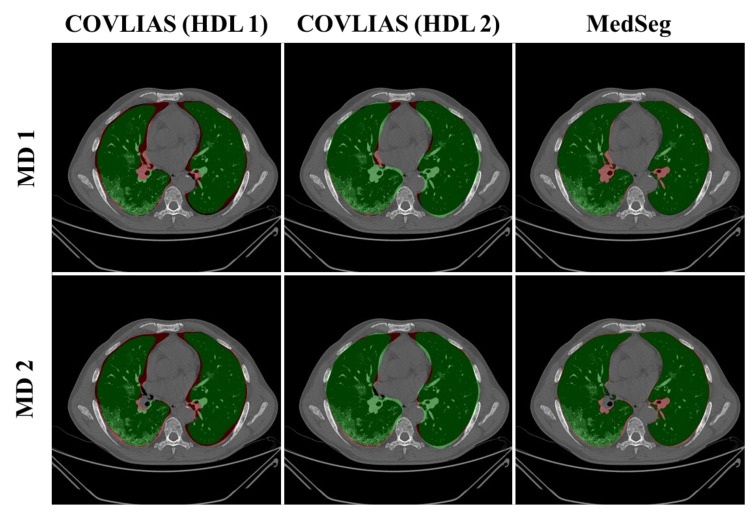
COVLIAS (HDL 1) (green) in column 1; COVLIAS (HDL 2) (green) in column 2; MedSeg (green) in column 3, MD 1 in row 1 (red); MD 2 in row 2 (red).

**Figure 10:**
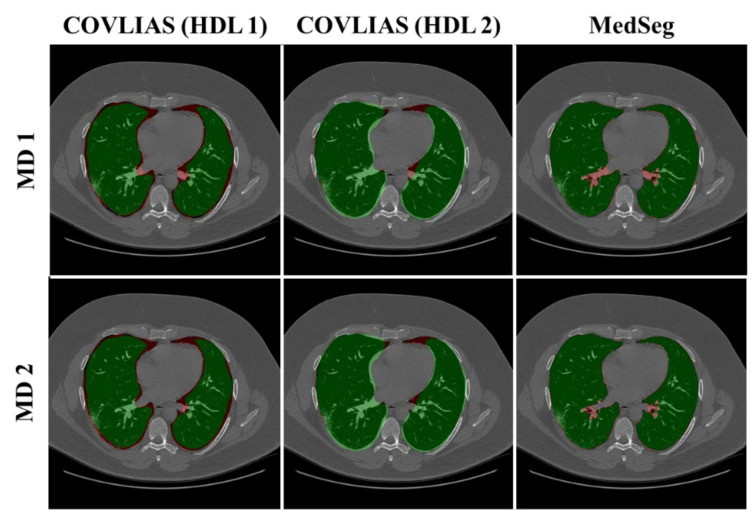
COVLIAS (HDL 1) (green) in column 1; COVLIAS (HDL 2) (green) in column 2; MedSeg (green) in column 3, MD 1 in row 1 (red); MD 2 in row 2 (red).

We present a comparison of CT lung segmentation for COVLIAS 1.0 vs. MedSeg using overlays of the binary mask from the two AI systems (see Figure 9 and Figure 10). Using a region-to-boundary convertor, the lung masks were then turned into lung boundary images, which were then placed over the original COVID-19 lung CT grayscale scans. We present (i) Bland–Altman (BA) plots, (ii) CC plots, (iii) receiver operating characteristic (ROC) curve, and (iv) Figure of Merit (FoM) as part of performance evaluation for COVLIAS and MedSeg against Manual Delineation (MD).

### 4.1. Performance: COVLIAS vs. MedSeg

**Figure 11:**
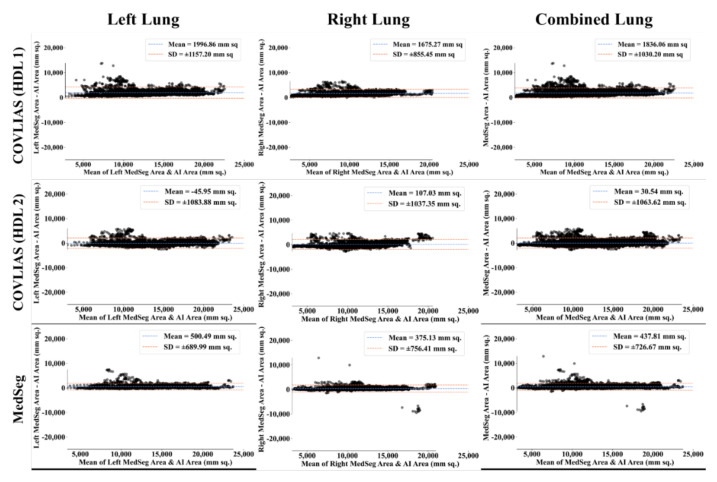
Bland–Altman plots: COVLIAS (row 1 and row 2) vs. MedSeg (row 3) using MD 1. Column 1: left lung, column 2: right lung, and column 3: mean of left and right. COVLIAS (HDL 1): VGG-SegNet; COVLIAS (HDL 2): ResNet-SegNet.

**Figure 12:**
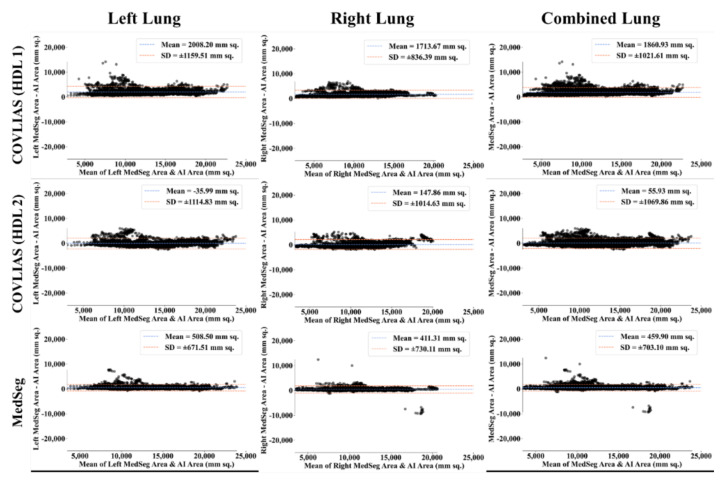
Bland–Altman plots: COVLIAS (row 1 and row 2) vs. MedSeg (row 3) using MD 2. Column 1: left lung, column 2: right lung, and column 3: mean of left and right. COVLIAS (HDL 1): VGG-SegNet; COVLIAS (HDL 2): ResNet-SegNet.

**Figure 13:**
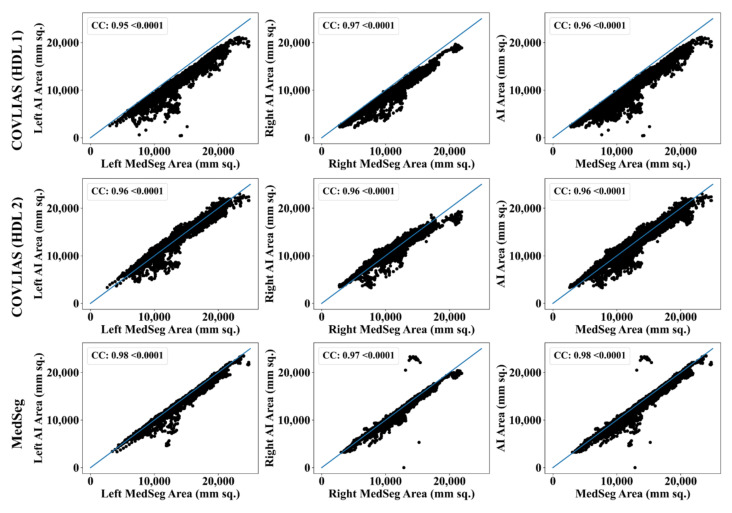
CC plots: COVLIAS (row 1 and row 2) vs. MedSeg (row 3) using MD 1. Column 1: left lung, column 2: right lung, and column 3: mean of left and right lungs. COVLIAS (HDL 1): VGG-SegNet; COVLIAS (HDL 2): ResNet-SegNet.

**Figure 14:**
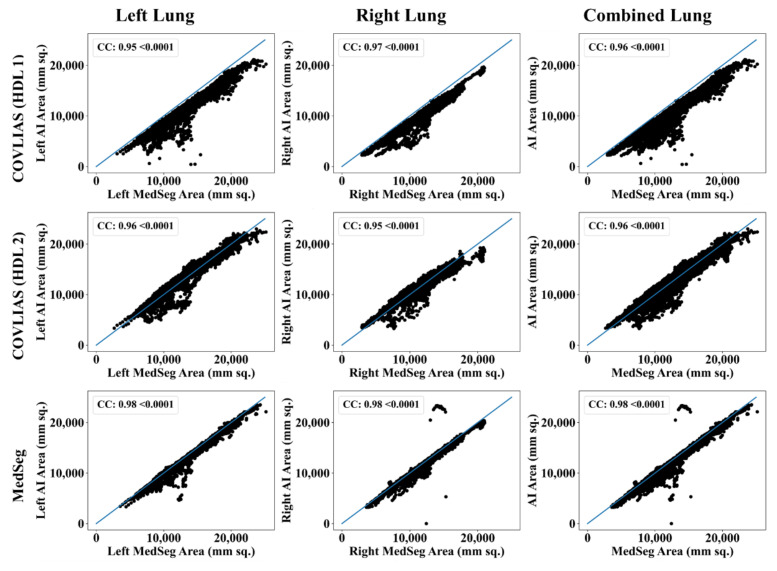
CC plots: COVLIAS (row 1 and row 2) vs. MedSeg (row 3) using MD 2. Column 1: left lung, column 2: right lung, and column 3: mean of left and right lungs. COVLIAS (HDL 1): VGG-SegNet; COVLIAS (HDL 2): ResNet-SegNet.

**Figure 15:**
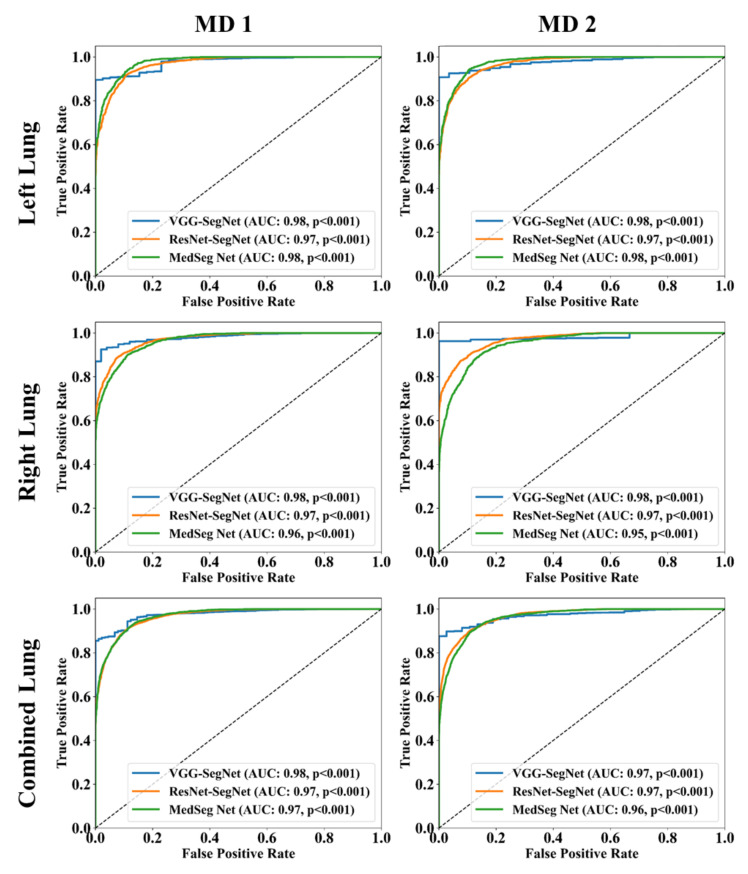
ROC plot: COVLIAS vs. MedSeg. Row 1: left lung, row 2: right lung, row 3: combined lung. Left: using MD 1, Right: using MD 2.

The Bland–Altman computation approach, based on our previous ideas [69,70], is used to show the consistency of two methods that uses the same variable. The mean and standard deviation of the lung area between the AI model of COVLIAS and MedSeg against MD region corresponding to MD 1 is shown in Figure 11 and Figure 12. Similarly, Figure 13 and Figure 14 show the CC plot for the AI model of COVLIAS and MedSeg against the MD 1 and MD 2, with the CC > 0.95 for all the AI models. A ROC curve shows how the diagnostic performance of an AI system changes as the discrimination threshold is changed. The ROC curve and AUC value for the three AI models are shown in Figure 15, with AUC > 0.95 for all three AI models. The figure of merit (FoM) is determined by the error’s statistical significance.

**Figure 16:**
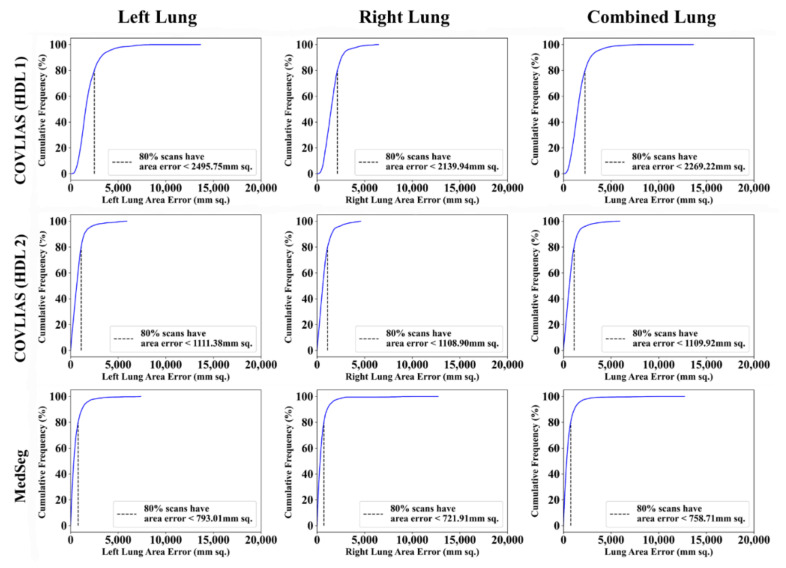
Cumulative frequency plots: COVLIAS (row 1 and row 2) vs. MedSeg (row 3) using MD 1. Column 1: left lung, column 2: right lung, and column 3: mean of left and right lungs. COVLIAS (HDL 1): VGG-SegNet; COVLIAS (HDL 2): ResNet-SegNet.

**Figure 17:**
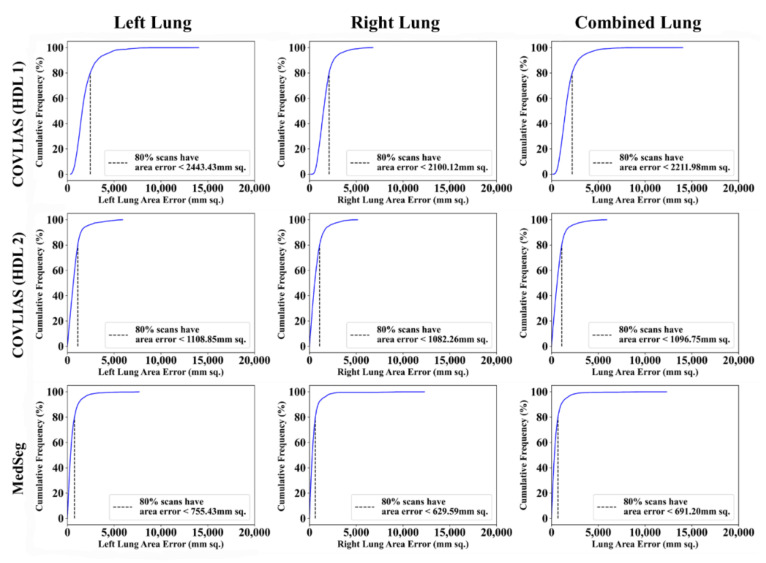
Cumulative frequency plots: COVLIAS (row 1 and row 2) vs. MedSeg (row 3) using MD 2. Column 1: left lung, column 2: right lung, and column 3: mean of left and right lungs. COVLIAS (HDL 1): VGG-SegNet; COVLIAS (HDL 2): ResNet-SegNet.

Cumulative frequency plot (Figure 16 and Figure 17) shows the lung area error for the AI model of COVLIAS and MedSeg against MD 1 and MD 2, with the 80% cutoff for all the AI models. Table 1 shows the values for Figure of Merit (FoM) and the percentage difference for the COVLIAS and MedSeg against the MD.

### 4.2. Statistical Tests

A standard Mann–Whitney, Paired *t*-Test, and Wilcoxon test were used to examine the system’s reliability and stability. When the distribution is not normal, the Wilcoxon test is employed instead of the paired *t*-test to assess if enough evidence supports the hypothesis. The statistical analysis was carried out using MedCalc software (Osteen, Belgium). We provided all conceivable combinations (six in total) for the COVLIAS and MedSeg against MD 1 and MD 2 to validate the system proposed in the study. The results of the Mann–Whitney, Paired *t*-Test, and Wilcoxon test are shown in Table 2.

### 4.3. Scientific Validation

**Figure 18:**
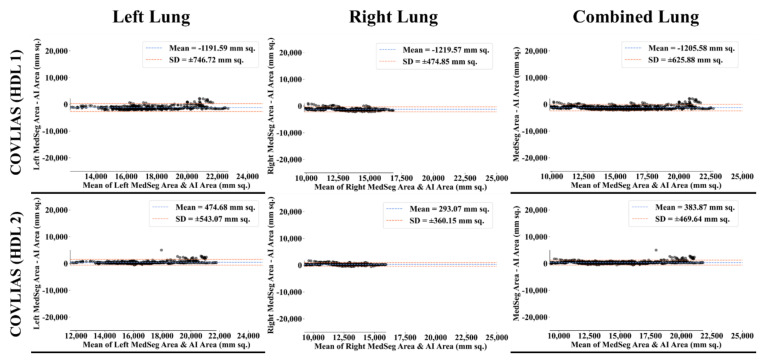
Bland–Altman plot: COVLIAS 1.0 vs. MedSeg. Left. Column 1: left lung, column 2: right lung, and column 3: combined lungs. COVLIAS (HDL 1): VGG-SegNet; COVLIAS (HDL 2): ResNet-SegNet.

**Figure 19:**
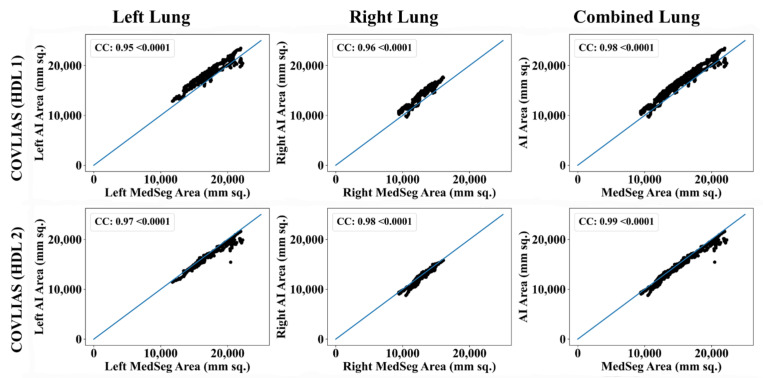
CC plot: COVLIAS 1.0 vs. MedSeg. Column 1: left lung, column 2: right lung, and column 3: combined lungs. COVLIAS (HDL 1): VGG-SegNet; COVLIAS (HDL 2): ResNet-SegNet.

**Figure 20:**
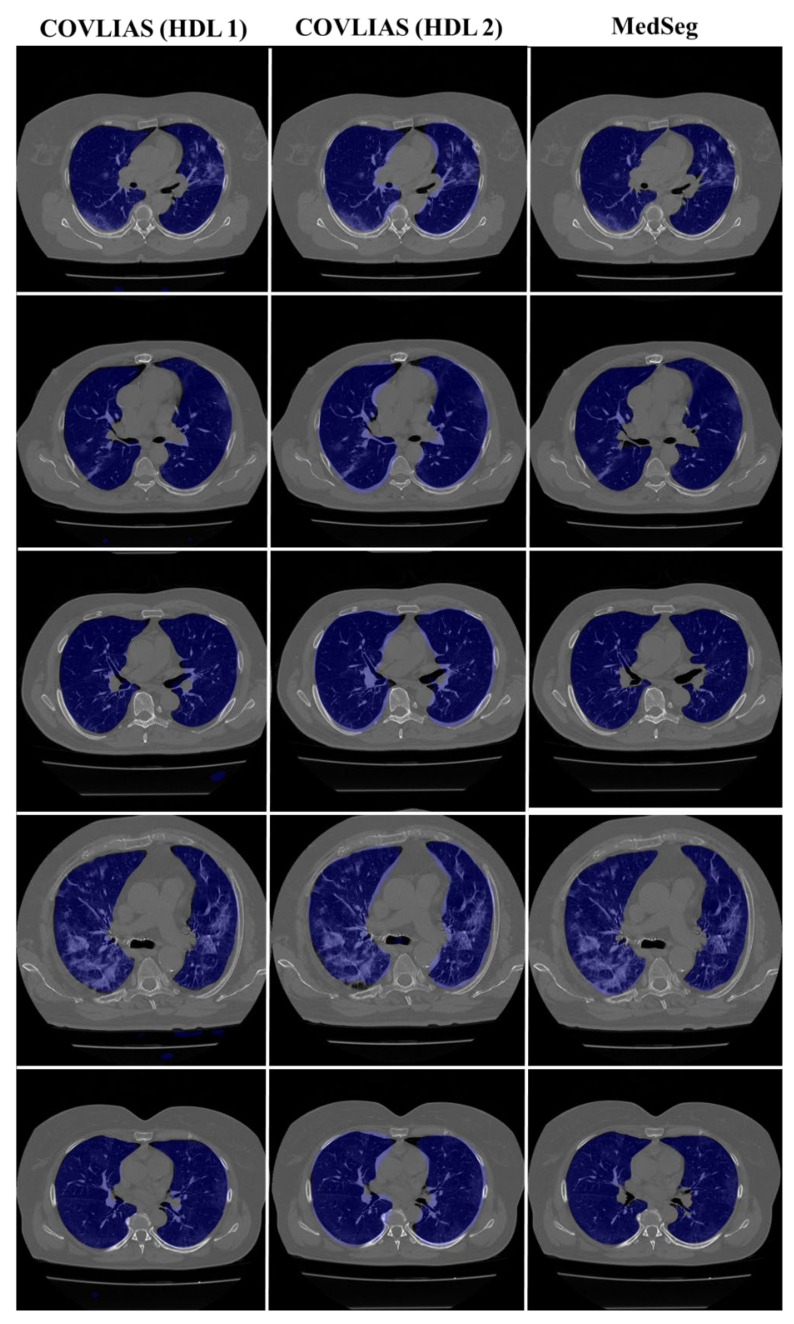
COVLIAS vs. MedSeg: Segmented mask (blue) on the raw CT CROATIA lung image.

To validate the COVLIAS system and benchmark against MedSeg, a validation cohort from CROATIA was used. This is part of the validation process, where the AI model in the COVLIAS was trained on the NOVARA (Italian) dataset consisting of 5000 CT lung images and validated on 500 CROATIAN CT lung images. Figure 18 shows BA plots for the COVLIAS vs. MedSeg using the CROATIAN data with a mean area error of ~1205 mm^2^ between COVLIAS (HDL 1) and MedSeg and ~383 mm^2^ between COVLIAS (HDL 2) and MedSeg, respectively. The mean CC (Figure 19) between COVLIAS (HDL 1) and MedSeg was 0.98 and between COVLIAS (HDL 2) and MedSeg was 0.99. Figure 20 shows the segmented binary lung overlay from the two AI systems COVLIAS and MedSeg on the raw CT lung image.

## 5. Discussion

**Figure 21:**
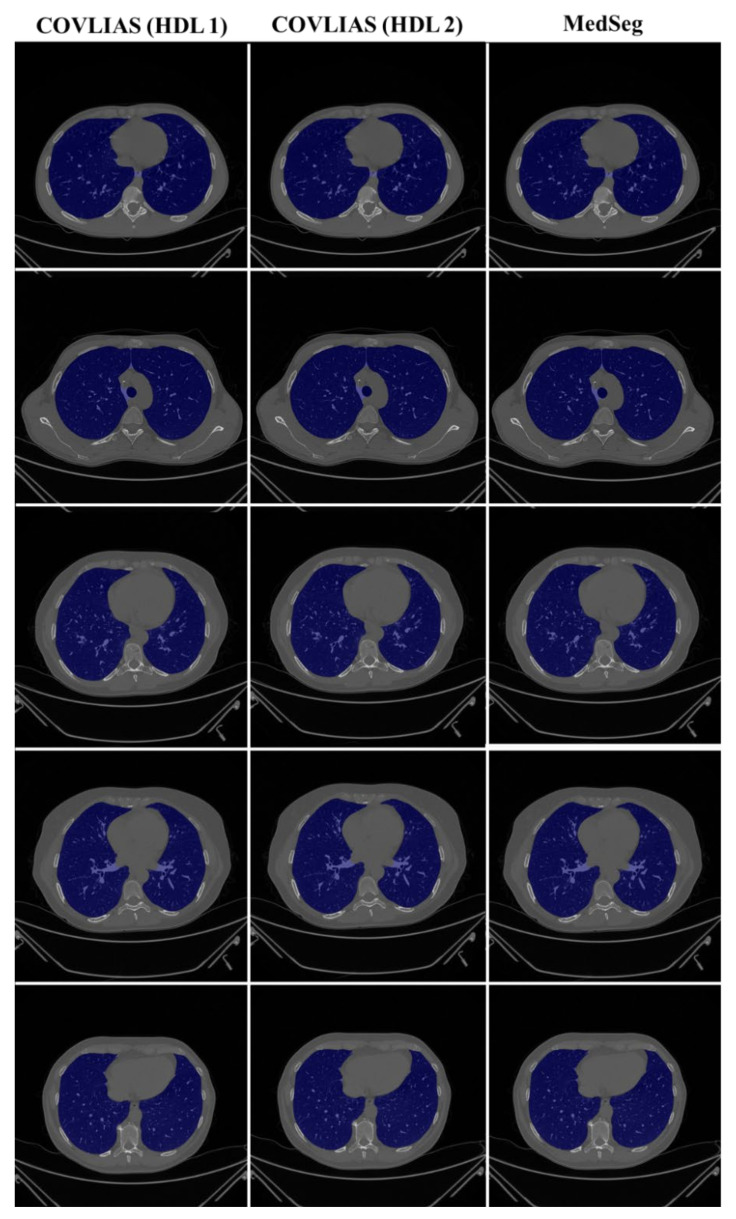
COVLIAS vs. MedSeg: Segmented mask (blue) on the Control CT lung image (large lung).

**Figure 22:**
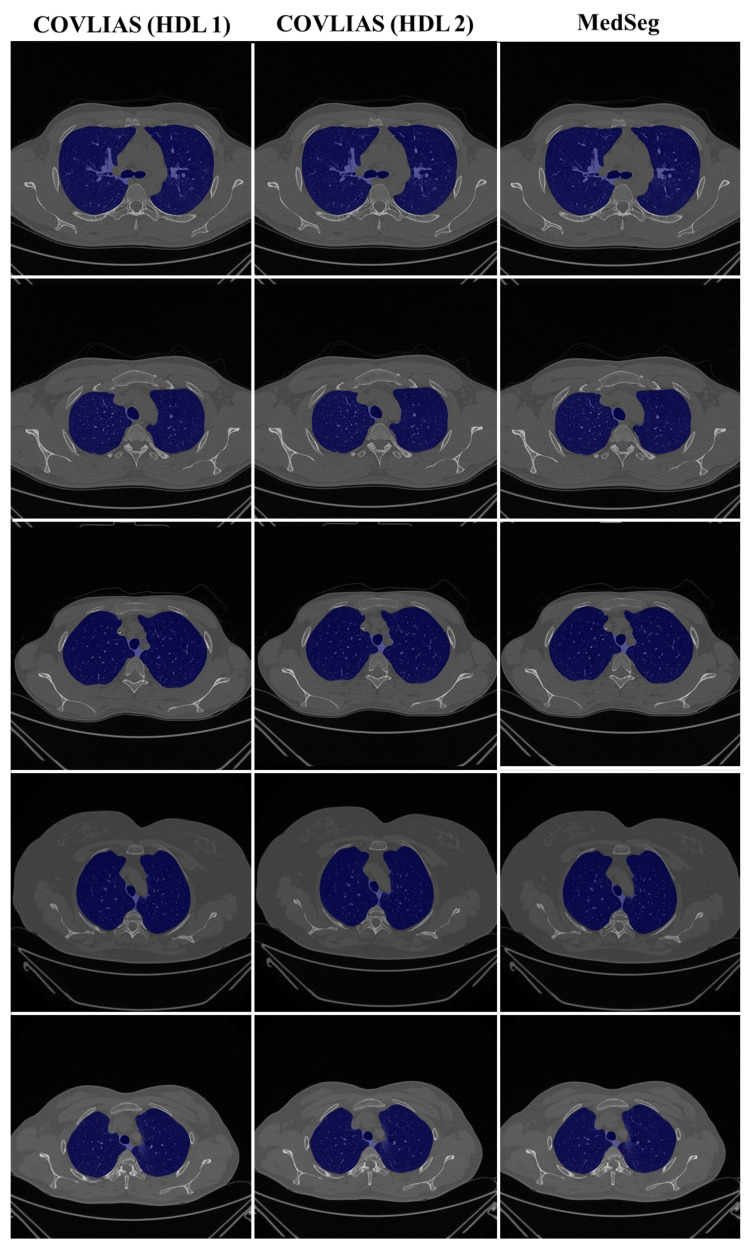
COVLIAS vs. MedSeg: Segmented mask (blue) on the Control CT lung image (small lung).

**Figure 23:**
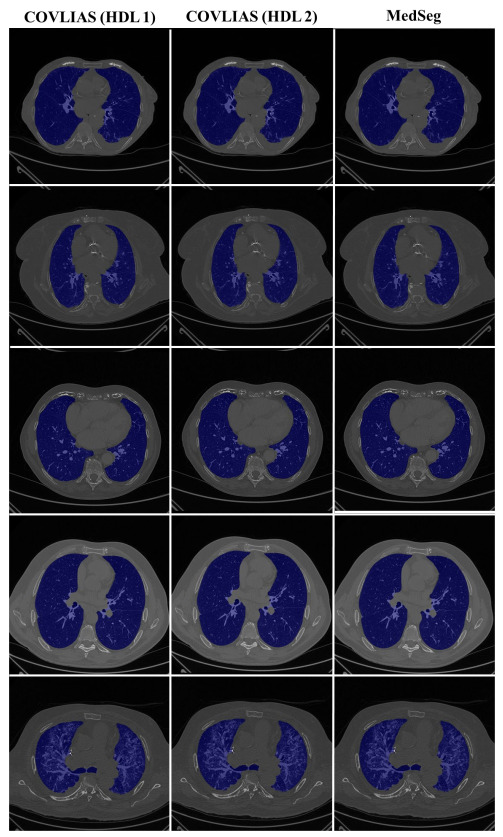
COVLIAS vs. MedSeg: Segmented mask (blue) on the non-COVID CT lung image (large lung).

**Figure 24:**
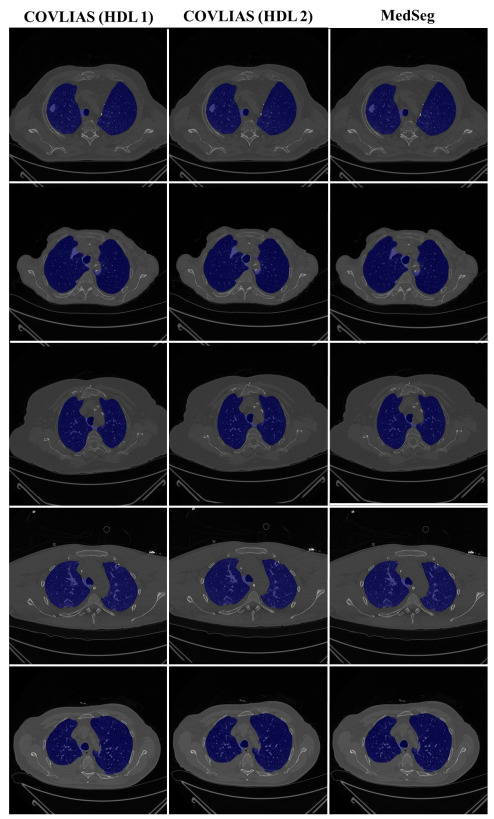
COVLIAS vs. MedSeg: Segmented mask (blue) on the non-COVID CT lung image (small lung).

The proposed study presents a brief comparison of two AI lung segmentation tools, COVLIAS 1.0 and MedSeg. COVLIAS has been trained on MD COVID-19 infected patient data from two MD using the K5 protocol [1]. We demonstrated the consistency in the AI system by using four performance evaluation metrics (i) BA plots, (ii) CC plots, (iii) ROC curve, and (iv) FoM. Figure 9 and Figure 10 show visual binary mask overlays, where red represents the MD lung, and the green represents the output of the AI model. It shows that ResNet-SegNet is precise in detecting the curves and portions that are missed by MedSeg and VGG-SegNet. Using the mean error of the combined lung area in Figure 11 and Figure 12, it shows that ResNet-SegNet gives the least error of 30.54 mm^2^ and 91.56 mm^2^ compared to 437.81 mm^2^ and 459.90 mm^2^ for MedSeg when using MD1 and MD2, respectively. COVLIAS 1.0 is an AI system that employed two HDL models (VGG-SegNet and ResNet-SegNet), while MedSeg used its own DL model. COVLIAS was designed to work on PNG, JPEG, DICOM, and NIfTI images, while MedSeg can only work on NIfTI [68] or DICOM [71] format images. Both COVLIAS and MedSeg processed the binary mask images to compute the lung area during performance evaluation. Figure 21 (large lung) and Figure 22 (small lung) shows the segmented mask on the control patients from ITALIAN CT lung dataset. Similarly, Figure 23 (large lung) and Figure 24 (small lung) shows the segmented mask on the non-COVID patients from the ITALIAN CT lung dataset.

### 5.1. A Special Note on MedSeg

MedSeg is a radiology-developed web-based segmentation tool for annotating CT/MRI images. You may instantly review and segment your photographs using your browser and your computer’s GPU without installing software or transferring your data to an external server. The basic requirements to run this tool are an up-to-date web browser, keyboard, mouse, and a GPU (for increasing the efficiency of segmentation). This tool accepts data in NIfTI [68] or DICOM [71] format. It is an interactive tool where the user can either drag and drop the image or select it from the directory. The next step is loading the CT thorax lung model for segmentation. MedSeg then gives the output in NIfTI format, which is then converted to PNG format. The performance evaluation (PE) metrics are computed using the PNG images. This includes (i) left and right lung separation, (ii) individual lung area calculation, and (iii) visual overlay generation.

### 5.2. Benchmarking

Various research using deep learning algorithms based on chest CT imaging to identify and segment COVID-19 instances from non-COVID-19 cases have been published [72,73,74,75]. However, most of the studies lack in individual lung area estimation, transparency overlay generation, and usage of HDL. Table 3 depicts the benchmarking table, which includes studies from Paluru et al. (2020) [76], Saood et al. (2021) [77], Cai et al. (2020) [78], Suri et al. (2021) [1], and Suri et al. (2021) [3], where [76,77,78] have used solo DL models to segment the lungs, compared to [1,3], where both solo DL and HDL model were used. This proposed study by Suri et al. consisted of two kinds of MD which are benchmarked against a web-based lung segmentation tool, MedSeg. Overall, this study offers intra-variability MD against COLVIAS and MedSeg for segmentation of COVID-19 based CT lung images.

### 5.3. Strength, Study limitation, and Extension

The experimental data set was used to compare COVLIAS and MedSeg. The results show proximity between COLVIAS and MedSeg. Unseen AI was conducted on validation data using COVLIAS and compared against MedSeg. The overall results showed COVLIAS and MedSeg having a 2.5% difference, meeting the industry standard of MedSeg. Intra-observer analysis was conducted during these comparisons. In spite of the encouraging results of COVLIAS on experimental and validation data sets, the pilot study can be enhanced by adding a bigger validation data set and conducting inter-observer analysis. Even though the COVLIAS is a well-balanced HDL system, the system can be implemented by taking the demographics and risk factors in a big data framework [79]. Several other models can be attempted in transfer learning or ensemble frameworks [4,56,57]. It would also be interesting to explore the segmentation of lungs with severe COVID-19 patients using the AI model. Our experimental and validation data GGO values were in the range 4.1 and 2. These are considered low to mild COVID GGO values. Since our AI models were trained on the mild COVID-19 CT scans, it is likely that we will be retraining the AI models should new COVID CT data have higher GGO values or have consolidations or crazy paving. There is the possibility that one might require different AI training models with a different intensity level of the COVID disease.

## 6. Conclusions

The study presented a segmentation comparison of two CT lung AI systems, namely, COVLIAS 1.0 (Global Biomedical Technologies, Inc., Roseville, CA, USA) and MedSeg. Our cohorts were taken from two nations, namely Italy (experimental data) and Croatia (validation data), having a sample size of 5500 CT scans. These cohorts were COVID mild, having a mean glass ground opacities of 4.1 and 2. Two AI-based HDL models of the COVLIAS, i.e., VGG-SegNet (19 layers, named HDL 1) and ResNet-SegNet (51 layers, named HDL 2), were used in the proposed study for benchmarking them against MedSeg. The error metrics for the two HDL systems, designed and developed using the two-gold standard manual delineations, were compared to validate the results from the AI systems. A trained radiologist annotated these manual delineations.

Our results on the experimental Italian data show that the CC between COVLIAS (HDL 1) and MD 1, COVLIAS (HDL 2) and MD 1, COVLIAS (HDL 1) and MD 2, and COVLIAS (HDL 2) and MD 2 were 0.96, 0.96, 0.96, and 0.96 with a mean of 0.96, respectively. The CC between (a) MedSeg and MD 1 and (b) MedSeg and MD 2 was 0.98 and 0.98, with the system’s mean 0.98. The difference in mean values between COVLIAS and MedSeg was in the range of 2.5%. On the validation (Croatia) data, our results show that the CC between (i) COVLIAS (HDL 1) and MedSeg and (ii) COVLIAS (HDL 2) and MedSeg was 0.98 and 0.99, respectively. As part of the validation, we also applied the two HDL training models to (a) non-COVID Italian and (b) Control Italian cohorts and compared them against MedSeg, demonstrating consistent lung segmentation results. To assess the system’s dependability and stability, a standard Mann–Whitney, Paired t-Test, and Wilcoxon tests were demonstrated. Our results showed clear evidence of comparable performance between COVLIAS 1.0 and MedSeg.

## Figures and Tables

**Table 1:** FoM table for COVLIAS and MedSeg for lung area error against MD.

	MD 1			MD 2			% Difference		
	Left	Right	Mean	Left	Right	Mean	Left	Right	Mean
MedSeg	96.42	96.85	96.61	96.36	96.55	96.45	0.1%	0.3%	0.2%
VGG-SegNet	92.45	93.41	92.89	92.40	93.13	92.73	0.1%	0.3%	0.2%
ResNet-SegNet	99.96	98.63	99.39	99.98	98.30	99.23	0.0%	0.3%	0.2%

**Table 2:** Mann–Whitney, Paired *t*-test, and Wilcoxon test for COVLIAS and MedSeg for combined lung area against MD.

	Mann-Whitney	Paired *t*-Test	Wilcoxon
COVLIAS (HDL 1) vs. MD 1	*p* < 0.0001	*p* < 0.0001	*p* < 0.0001
COVLIAS (HDL 1) vs. MD 2	*p* < 0.0001	*p* < 0.0001	*p* < 0.0001
COVLIAS (HDL 2) vs. MD 1	*p* < 0.0001	*p* < 0.0001	*p* < 0.0001
COVLIAS (HDL 2) vs. MD 2	*p* < 0.0001	*p* < 0.0001	*p* < 0.0001
MedSeg vs. MD 1	*p* < 0.0001	*p* < 0.0001	*p* < 0.0001
MedSeg vs. MD 2	*p* < 0.0001	*p* < 0.0001	*p* < 0.0001

**Table 3:** Benchmarking table.

Author (Year)	# of Patients	Gender	# of Images	# of Tracers	Variability Studies	Image Size	Comparison	Model	Solo vs. HDL	Modality	Area Error
Cai et al. (2020) [78]	99	58 males; 41 females	6336	2	✗	-	-	UNet	Solo	2D	✗
Paluru et al. (2021) [76]	69	-	4339	NA	✓	512	7	Anam-net	Solo	2D	✗
Saood et al. (2021) [77]		-	100	NA	✗	256	2	UNet, SegNet	Solo	2D	✗
Suri et al. (2021) [1]	72	46 males; 26 females	5000	1	✗	768	4	NIH, SegNet, VGG-SegNet, ResNet-SegNet	Both	2D	✓
Suri et al. (2021) [3]	72	46 males; 26 females	5000	2	✓	768	13	PSP Net, VGG-SegNet, ResNet-SegNet	Both	2D	✓
Suri et al. (2021) Proposed	79	51 males. 28 females	5500	1	✓	768	4	VGG-SegNet, ResNet-SegNet, MedSeg	HDL	2D	✓

#: number; HDL: Hybrid Deep Learning.
